# Pediatric Outcomes of Emergency Medical Services Non-Transport Before and During the COVID-19 Pandemic

**DOI:** 10.5811/westjem.18408

**Published:** 2024-02-09

**Authors:** Lori Pandya, Brandon Morshedi, Brian Miller, Halim Hennes, Mohamed Badawy

**Affiliations:** * University of Texas Southwestern Medical Center, Department of Pediatrics, Division of Emergency Medicine, Dallas, Texas; † University of Arkansas for Medical Sciences, Deprtment of Emergency Medicine, Little Rock, Arkansas; ‡ University of Texas Southwestern Medical Center, Department of Emergency Medicine, Dallas, Texas

## Abstract

**Introduction:**

Pediatric patients account for 6–10% of emergency medical services (EMS) activations in the United States. Approximately 30% of these children are not transported to an emergency department (ED). Adult data in the literature reports higher hospitalization and complications following non-transport. Few studies discuss epidemiology and characteristics of pediatric non-transport; however, data on outcome is limited. Our primary aim was to determine outcomes of non-transported children within our urban EMS system before and during the COVID-19 pandemic. Our secondary objective was to explore reasons for non-transport.

**Methods:**

This was a prospective, descriptive pilot study. We compared EMS data for September 2019 (pre-COVID-19) to September 2020 (pandemic). Included were children aged 0–17 years who activated EMS and did not receive transport to the primary hospital for the EMS capture area. We defined outcomes as repeat EMS activation, ED visits, and hospital admissions, all within 72 hours. Data was obtained via electronic capture. We used descriptive statistics to analyze our data, chi square for categorical data, stepwise logistic regression, and univariate logistic regression to test for association of covariates with non-transport.

**Results:**

There were 1,089 pediatric EMS activations in September 2019 and 780 in September 2020. Non-transport occurred in 633 (58%) in September 2019 and 412 (53%) in September 2020. Emergency medical services was reactivated within 72 hours in the following: 9/633 (1.4%) in 2019; and 5/412 (1.2%) in 2020 (*P* = 0.77). Visits to the ED occurred in 57/633 (9%) in 2019 and 42/412 (10%) in 2020 (*P* = 0.53). Hospital admissions occurred in 10/633 (1.5%) in 2019 and 4/412 (0.97%) in 2020 (*P* = 0.19). One non-transported patient was admitted to the intensive care unit in September 2020 (<1%) and survived. Hispanic ethnicity, age >12 years, and fever were associated with repeat EMS activation. The most common reason for non-transport in both study periods was that the parent felt an ambulance was not necessary (47%).

**Conclusion:**

In our system, non-transport of pediatric patients occurred in >50% of EMS activations with no significant adverse outcome. Age >12 years, fever, and Hispanic ethnicity were more common in repeated EMS activations. The most common reason for non-transport was parents feeling it was not necessary. Future studies are needed to develop reliable EMS guidelines for pediatric non-transport.

Population Health Research Capsule What do we already know about this issue?

*Up to 30% of pediatric EMS activations are not transported to an ED. Adult data reports adverse outcomes following non-transport; pediatric data is limited.*

What was the research question?

*We aimed to determine outcomes of non-transported children within our EMS system before and during COVID-19.*

What was the major finding of the study?

*There was no difference in outcomes pre/during COVID-19: EMS reactivation (1.3% of all patients) (P = 0.77); ED visits (P = 0.53); and admission (P = 0.19).*

How does this improve population health?

*Future studies are needed to develop reliable guidelines for pediatric non-transport, which could decrease burden on the medical system especially during pandemics.*

## INTRODUCTION

Pediatric patients historically account for up to 10% of emergency medical services (EMS) activations in the United States,^1^ with more recent literature suggesting 6%.^2^ A national EMS data review noted that 30% of pediatric patients are not transported to a medical facility for further evaluation and care.^2^ ^–^ ^6^ The reasons for non-transport are broad, including factors such as parental refusal and type of complaint (ie, musculoskeletal trauma, respiratory illness). While data exists regarding rates of and factors related to pediatric EMS non-transport, outcomes are limited to a few studies. In the adult literature, non-transport was associated with a 16% hospitalization rate^7^ and in some cases serious or fatal outcomes.^8^ One pediatric study noted non-transported patients <3 years of age were 1.3 times more likely to have a subsequent emergency department (ED) visit,^9^ while another reported a 10% hospitalization rate after pediatric non-transport for parental refusal.^10^ During the COVID-19 pandemic, data suggests that EMS call volumes and non-transport rates changed, with a decline in overall EMS response volumes and an increase in the rate of non-transports.^11^ Little is known about whether this impacted outcomes for children who were non-transported.

Our primary objective was to determine pediatric outcomes of non-transport within our large EMS system before and during the COVID-19 pandemic. Outcomes were defined as repeat EMS activation, in-person ED visits, and/or hospital admissions, all within 72 hours of initial EMS activation. We also aimed to describe demographic factors associated with subsequently needing medical attention after EMS non-transport. A secondary objective was to identify reasons for non-transport within our system both pre- and during the COVID-19 pandemic. We chose to compare pre- and during the pandemic to determine whether there was a change in utilization or in EMS clinicians’/parents’ behavior during a pandemic to better prepare our systems for the future.

## MATERIALS AND METHODS

### Study Setting

This was a prospective, descriptive pilot study at a large, urban, fire-based EMS system in the City of Dallas, Texas, with 59 stations and ∼1,800 EMS responders serving a total population of 1.3 million, with approximately 25% of that population <18 years of age. The study was approved by the institutional review board.

### Inclusion of Patients

We included children aged 0–17 years with EMS activation who did not receive EMS transport during the study period. We selected two one-month time periods, September 2019 (pre-COVID-19) and September 2020 (COVID-19 pandemic). During the study period, all non-transports of pediatric patients were, per protocol, required to have online medical control (OLMC) consultation and/or audio recording. Audio-recorded refusal was obtained via handheld tablet using a standardized script. Any EMS-initiated non-transports were not allowed in the system, and all non-transports were initiated by the parent or guardian.

### Data Acquisition

We obtained and compared EMS data through comprehensive manual review of the prehospital electronic health record (EHR) from a daily automated report of the two periods. The EMS records were electronically matched using name and date of birth (DOB) for repeat EMS activation within 72 hours. At our pediatric hospital health system, which is the primary tertiary care children’s hospital for the EMS capture area, we queried the EHR for ED visits and hospital admissions within 72 hours of EMS activation using the same name and DOB. If concerns arose for a name spelling error, we used DOB and address to confirm an identity match. Demographic data, chief/dispatch complaint, EMS vitals, non-transport volume, and non-transport reason were manually abstracted from our EMS electronic patient care database/automated report (by either the principal investigator PI or a single, trained research assistant [RA]). Race/ethnicity was EMS identified using a drop-down menu in the electronic patient care record; the categories are per NEMSIS (National EMS Information System). Because prior versions of NEMSIS combined race and ethnicity there are not separate fields. In-person ED visits and hospital admissions (including inpatient observation and intensive care unit [ICU] admission) within 72 hours of EMS activation and final disposition (discharge vs death) were manually abstracted from the hospital health system EHR (by either the PI or a single trained RA). This included hospital presentations after refusal that came by repeat EMS activation and other means (eg, private vehicle).

### Outcomes

We defined primary outcomes as repeat EMS activation, in-person ED visits, and/or hospital admissions, all within 72 hours of initial EMS activation. We used the 72-hour follow-up window based on other published papers in this area.^12^ ^–^ ^16^ Pediatric EMS protocols did not change between these two study periods. The population was stratified by age group (similar to previously published studies^2^ ^,^ ^17^ ^,^ ^18^) and chief complaint to determine whether there was a higher proportion of non-transport based on age and the most common non-transport diagnosis. We classified EMS chief complaint/diagnosis into the following categories: fever; gastrointestinal; respiratory; trauma; neurological; pain; mental health; and other. Reason for parental refusal of transport was described (EMS documented).

### Analysis

We analyzed categorical data using the chi-squared test. The Fisher exact test was used for smaller sample sizes (ie, hospital and ICU admission data). We used the *t*-test and Wilcoxon rank-sum test for EMS vital signs. Initial EMS vital signs of temperature, heart rate, respiratory rate and oxygen saturation were abstracted for each subject and defined as abnormal based on normal age-related ranges within the Pediatric Advanced Life Support guidelines.^19^ We made correction for multiple testing and used only cases with complete data in the final analysis. Covariates for analysis were a priori based on previous literature. To identify covariates’ association with the outcome we performed a stepwise logistic regression. However, the analysis identified only one covariate, and we used a univariate logistic regression to test for association of that covariate within non-transport outcomes. We did not calculate a predetermined sample size, as this was a pilot study. Results are presented as odds ratios with 95% confidence intervals (CI), taking *P*-values of <0.05 as significant. Statistical analyses were performed using SAS for Windows release 9.4 (SAS Institute, Inc., Cary, NC).

## RESULTS

Annual pediatric EMS volumes were 12,663 (2019) and 10,429 (2020). There were 1,089 pediatric EMS activations in September 2019 vs 780 activations in September 2020 (Figure). Non-transport occurred in 633 (58%) activations in September 2019 vs 412 (53%) in September 2020 (Table 1). Per our EMS protocol, we obtained OLMC and/or audio recording in 84% of non-transports. Demographics are listed in Table 2.

**Figure.**
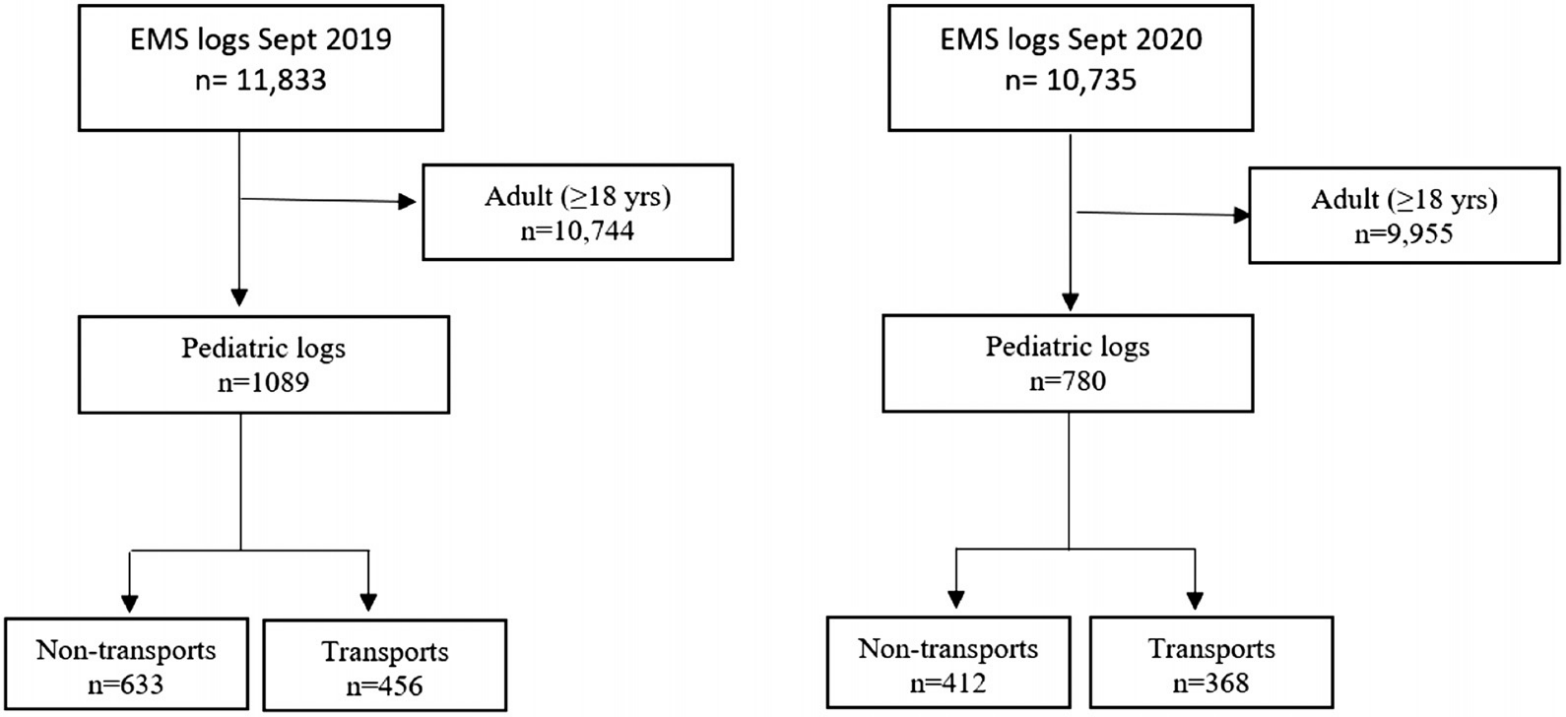
STROBE diagram illustrating patient inclusion. *EMS*, emergency medical services.

**Table 1.** Volume of non-transported pediatric patients before (2019) and during (2020) the COVID-19 pandemic.

	Sept 2019	Sept 2020
EMS activations	1089	780
Non-transport (%)	633 (58)	412 (53)

**Table 2.** Demographics of non-transported pediatric patients before (2019) and during (2020) the COVID-19 pandemic.

	2019 (N = 633)	2020 (N = 412)	All (N = 1,045)	*P*-value
Patient Age (mos)	113 (38.0–182.0)	111.5 (33.4–181.5)		
Patient Age (yrs)				0.9
0–2	121 (19.2%)	76 (18.6%)	197 (19.0%)	
2–5	107 (17.0%)	63 (15.4%)	170 (16.4%)	
5–12	158 (25.1%)	105 (25.7%)	263 (25.3%)	
>=12	244 (38.7%)	164 (40.2%)	408 (39.3%)	
Gender				0.7
Female	312 (49.5%)	208 (50.6%)	520 (50.0%)	
Male	318 (50.5%)	203 (49.4%)	521 (50.0%)	
Race				0.7
Black or African American	337 (53.9%)	207 (50.9%)	544 (52.7%)	
White	63 (10.1%)	38 (9.3%)	101 (9.8%)	
Hispanic or Latino	205 (32.8%)	149 (36.9%)	354 (34.3%)	
Other	20 (3.2%)	13 (3.2%)	33 (3.2%)	

Patient age noted as mean with interquartile range.

### Primary Outcomes

During September 2019 EMS was reactivated within 72 hours in 9/633 (1.4%) activations, ED visits occurred within 72 hours in 57/633 cases (9%), and hospital admissions occurred in 10/633 (1.5%). During September 2020, EMS was reactivated within 72 hours in 5/412 (1.2%) activations, ED visits occurred within 72 hours in 42/412 cases (10%), and hospital admissions occurred in 4/412 (1%). One non-transported patient was subsequently admitted to the ICU in September 2020 (<1%) and survived to discharge. There were no statistical differences in outcomes of non-transport pre- and during the pandemic (Table 3).

**Table 3.** Outcomes of non-transported pediatric patients before (2019) and during (2020) the COVID-19 pandemic.

Outcomes of non-transport (Within 72 hrs)	2019 (N = 633)	2020 (N = 412)	All (N = 1,045)	*P*-value
Repeat EMS activation	9 (1.4%)	5 (1.2%)	14 (1.3%)	0.8
Transport to ED on repeat activation	9 (1.4%)	4 (1.0%)	13 (1.2%)	0.5
ED visit	57 (9.0%)	42 (10.2%)	99 (9.5%)	0.5
Inpatient hospital admission	10 (1.6%)	4 (1%)	14 (1.3%)	0.2
ICU during hospital admission	0 (0.0)	1 (0.2)	1 (0.1)	0.4

### Secondary Outcomes

We further analyzed non-transport outcomes in September 2020 to determine whether there was a higher proportion of non-transport related to specific variables (gender, race/ethnicity, age, EMS diagnosis, and vital signs). Our percentage of missing variables ranged from 1–15%; however, in the analysis we used only cases with complete data. In those children who had repeat EMS activation within 72 hours, Hispanic ethnicity, age >12 years, and fever on EMS vitals were statistically significant factors for repeat activations. There was no difference in gender, EMS diagnosis, heart rate, respiratory rate, or oxygen saturation (Table 4). For those children with an EMS reactivation resulting in transport to the ED, a diagnosis of trauma, Hispanic ethnicity, age >12 years, and fever were significant (Table 5). Of those children with an ED visit within 72 hours of EMS non-transport, male gender was the only significant variable. There was no difference in race/ethnicity, age, diagnosis, or vital signs, including temperature (Table 6).

**Table 4.** Non-transported outcome during the COVID-19 pandemic- repeat EMS activation.

Variable	Category	Odds Ratio	95% C.I. for Odds Ratio	*P*-value
Gender	Female			
	Male	1.310	(0.468, 3.668)	0.60
Race/Ethnicity	Black/African American			
	White	0.194	(0.011, 3.334)	0.25
	Hispanic	0.167	(0.031 ,0.909)	0.03
	Other	0.588	(0.033, 10.526)	0.71
Patient age (yrs)	0–2			
	2–5	0.225	(0.038, 1.320)	0.09
	5–12	0.341	(0.094, 1.234)	0.10
	>=12	0.219	(0.061, 0.790)	0.02
EMS chief complaint/diagnosis	Fever			
	Gastrointestinal	0.527	(0.073, 3.799)	0.52
	Mental Health	2.414	(0.293, 19.912)	0.41
	Neurological	0.288	(0.041, 2.043)	0.21
	Other	0.206	(0.052, 0.817)	0.02
	Pain	0.128	(0.006, 2.610)	0.18
	Respiratory	0.225	(0.043, 1.187)	0.07
	Trauma	0.108	(0.016, 0.758)	0.02
EMS vital signs				
	Temp	2.645	(1.007, 6.943)	0.04
	HR	1.007	(0.979, 1.036)	0.60
	RR	1.023	(0.991, 1.057)	0.16
	Sat	0.966	(0.920, 1.015)	0.17

*Temp*, temperature; *HR*, heart rate; *RR*, respiratory rate; *Sat*, oxygen saturation.

**Table 5.** Non-transported outcome during the COVID-19 pandemic- transported to ED on repeat EMS activation.

Variable	Category	Odds Ratio	95% C.I. for Odds Ratio	*P*-value
Gender	Female			
	Male	1.645	(0.219, 1.899)	0.42
Race/Ethnicity	Black/African American			
	White	4.765	(0.276, 82.237)	0.28
	Hispanic	5.532	(1.009, 30.320)	0.04
	Other	1.573	(0.087, 28.284)	0.75
Patient age (yrs)	0–2			
	2–5	3.835	(0.639, 23.029)	0.14
	5–12	2.526	(0.678, 9.415)	0.16
	>=12	3.932	(1.058, 14.618)	0.04
EMS chief complaint/diagnosis	Fever			
	Gastrointestinal	1.897	(0.263, 13.667)	0.52
	Mental Health	0.414	(0.050, 3.417)	0.41
	Neurological	3.467	(0.0489, 24.553)	0.21
	Other	5.952	(1.419, 24.965)	0.01
	Pain	7.784	(0.383, 158.149)	0.18
	Respiratory	4.435	(0.842, 23.346)	0.07
	Trauma	9.223	(1.319, 64.478)	0.02
EMS vital signs				
	Temp	0.378	(0.144, 0.993)	0.04
	HR	1.001	(0.971, 1.032)	0.92
	RR	0.977	(0.947, 1.009)	0.16
	Sat	1.035	(0.985, 1.088)	0.17

*Temp*, temperature; *HR*, heart rate; *RR*, respiratory rate; *Sat*, oxygen saturation.

**Table 6.** Non-transported outcome during the COVID-19 pandemic- ED visit within 72 hours.

Variable	Category	Odds Ratio	95% C.I. for Odds Ratio	*P*-value
Gender	Female			
	Male	1.595	(1.047, 2.430)	0.02
Race/Ethnicity	Black/African American			
	White	0.684	(0.292, 1.602)	0.38
	Hispanic	1.357	(0.879, 2.096)	0.16
	Other	0.459	(0.085, 2.481)	0.36
Patient age (yrs)	0–2			
	2–5	1.215	(0.604, 2.446)	0.58
	5–12	1.298	(0.695, 2.421)	0.41
	>=12	1.011	(0.556, 1.838)	0.97
EMS chief complaint/diagnosis	Fever			
	Gastrointestinal	0.919	(0.280, 3.018)	0.88
	Mental Health	1.939	(0.365, 10.301)	0.43
	Neurological	0.877	(0.313, 2.460)	0.80
	Other	0.589	(0.251, 1.380)	0.22
	Pain	0.545	(0.158, 1.885)	0.33
	Respiratory	0.654	(0.257, 1.665)	0.37
	Trauma	0.858	(0.355, 2.073)	0.73
EMS vital signs				
	Temp	0.983	(0.679, 1.424)	0.92
	HR	1.009	(0.998, 1.020)	0.10
	RR	1.004	(0.977, 1.032)	0.76
	Sat	0.982	(0.940, 1.027)	0.42

*Temp*, temperature; *HR*, heart rate; *RR*, respiratory rate; *Sat*, oxygen saturation.

### Race/Ethnicity

In our large, urban county in 2020, Black residents made up 22.8% of the total population^20^ and accounted for approximately 53% of all pediatric EMS activations during our study month. Of all non-transported children in our study month 50% were Black. Hispanic/Latino accounted for 41% of the total population and 34% of all pediatric activations; 36% of pediatric non-transports were identified as Hispanic/Latino. In our urban county, 27% of the total population identified as White and made up 10% percent of total pediatric activations. Of those non-transported children 9% were White.

### Reasons for Non-transport

In the pre-pandemic period (September 2019), the reason for non-transport was filed for 354 (55%) of activations as follows: parent felt ambulance not necessary (47.7%); chief complaint resolved (24.9%); transport by private vehicle (20%); and other (3.1 %). In September 2020, the reason for non-transport was documented in 207 (49%) cases, with the most common reason being parent felt ambulance was not necessary (58%); followed by transport by private vehicle (22.2%); chief complaint resolved (15.5%); and other (4.8%).

## DISCUSSION

We found our rates of pediatric non-transport (both pre-and during pandemic) to be higher than the previously reported 16.3%–30.1%.^2^ ^–^ ^6^ Despite the higher rate of non-transport, our pediatric outcomes were favorable. The EMS reactivation and hospital admissions occurred in less than 1.5% of those children not transported to a healthcare facility. During our selected month in the pandemic, only one patient (<1%) required ICU care and survived to hospital discharge. Visits to the ED within 72 hours occurred in approximately 10% of children not transported; further study is needed to evaluate this subset of patients.

A recent published study from the United Kingdom showed a similar rate of pediatric EMS reactivation (2%) after ambulance non-transport. Subsequent ED visits were higher than in our findings (up to 24%), and hospital admissions were also higher (as high as 6% compared to our 1.5%). As in our study, no deaths occurred in pediatric non-transport.^16^ Another study showed approximately 14% ED visits after non-transport, <1% hospital admission, and again no deaths.^21^ A Scandinavian study reported 17.4% of non-transported children visited the ED, although this was within 96 hours compared to our 72-hour timeframe. Two patients were admitted to the ICU (compared to one in our study), and again no deaths occurred.^22^

All primary outcomes were not significantly different when compared to pre-pandemic data. Of note, we used the 72-hour follow-up window based on other published papers in this area,^12^ ^–^ ^16^ while acknowledging the balance between a longer window catching more cases but increasing the risk that those are not related to the index visit.

The majority of non-transported children were Black (50%); however, this was expected based on our demographics (the majority of all pediatric EMS activations during our study month were Black). Similarly, Hispanic/Latino children accounted for 34% of pediatric EMS activations and 36% of non-transports. This finding differs from prior studies that show a lower rate of non-transport for Black^3^ and Hispanic^6^ children. Our study is similar to a recent, large national study by Ward et al, which showed no association of race/ethnicity with non-transport.^2^

Although we found no association with race/ethnicity for non-transport, Hispanic children in our study were more likely to have repeat EMS activations within 72 hours. Age >12 years old and documented fever were also associated with repeat EMS activations. This age association with repeat activations may be due to a lower overall rate of non-transport in younger kids, both in our study and others^6^ and the postulated lack of EMS responders’ comfort level assessing young children.^14^ We also found that chief complaint/diagnosis was not significantly related to EMS non-transport during the pandemic, although children with trauma were not surprisingly transported more often to the ED if EMS was reactivated within 72 hours. Interestingly, EMS vitals (except fever) did not seem to play a role in our primary outcomes.

In our study we observed no significant difference in the percentage or outcomes of pediatric non-transport during the COVID-19 pandemic compared to pre-pandemic. It is important to note that EMS protocols did not change between these two study periods. While many EMS agencies adopted more permissive “non-transport” policies in anticipation of higher EMS call volumes and 9-1-1 overuse for minor, flu-like illness symptoms, our system did not adopt any such policy; thus, it is a truer comparison.

Our reasons for non-transport are similar to those previously reported in the literature.^5^ ^–^ ^7^ ^,^ ^23^ During the pandemic, there was approximately a 10% increase in “parents feel an ambulance is not necessary.” It is unclear whether this was directly related to the pandemic and fear of COVID-19 exposure or to missing data.

## LIMITATIONS

There are certain limitations of this study. Although we are the primary children’s hospital and urgent care within the jurisdiction served by the EMS system, there was the potential to miss repeat ED visits at a non-affiliated adult ED/urgent care. Future studies will include a phone call follow-up with the patient/family. We selected a single month, due to our high volumes, for this pilot study, assuming it would be representative of other months. Data was obtained through manual review of prehospital electronic patient care records obtained from an automated report, resulting in some occasional incomplete data. Hospital records (ED and inpatient) were matched using name and DOB, potentially missing subjects if there was an error in name spelling or provided DOB. If concerns arose for a mismatch, the provided address was used to confirm an identity, but this data was not always available. Furthermore, the EMS system’s clinical practice guidelines (protocols) require consultation with online medical control for patients <18 years old and for specific conditions and vital sign parameters. In this study we did not examine the proportion of non-transported patients with online medical control actually contacted. It is not known whether this influenced the safety of non-transports. Lastly, reason for non-transport was missing in up to 50% of data, and the reason was as documented by the EMS clinician.

## CONCLUSION

In our system, non-transport of pediatric patients occurred in over 50% of EMS activation with no significant adverse outcome. The most common reason for non-transport was parents feeling it was not necessary. Age >12 years, presence of fever, and Hispanic ethnicity were more common in repeated EMS activations. Chief complaint/diagnosis did not seem to play a role in repeat EMS activations or subsequent ED visits after non-transport. We observed no significant difference in the percentage or outcomes of pediatric non-transport during the COVID-19 pandemic compared to pre-pandemic. Additional studies are needed to develop reliable EMS guidelines for pediatric non-transport.

## References

[1] Diggs LA Sheth-Chandra M De Leo G . Epidemiology of pediatric prehospital Basic Life Support care in the United States. Prehosp Emerg Care. 2016;20(2):230–8. 26555372 10.3109/10903127.2015.1076099

[2] Ward C Zhang A Brown K et al . National characteristics of non-transported children by emergency medical services in the United States. Prehosp Emerg Care. 2022;26(4):537–46. 34570670 10.1080/10903127.2021.1985666 PMC9061893

[3] Ramgopal S Owusu-Ansah S Martin-Gill C . Factors associated with pediatric nontransport in a large emergency medical services system. Acad Emerg Med. 2018;25(12):1433–41. 30370989 10.1111/acem.13652

[4] Richard J Osmond MH Nesbitt L et al . Management and outcomes of pediatric patients transported by emergency medical services in a Canadian prehospital system. CJEM, 2006;8(1):6–12. 17175623 10.1017/s1481803500013312

[5] Kannikeswaran N Mahajan PV Dunne RB et al . Epidemiology of pediatric transports and non-transports in an urban emergency medical services system. Prehosp Emerg Care. 2007;11(4):403–7. 17907024 10.1080/10903120701536677

[6] Gerlacher GR Sirbaugh PE Macias CG . Prehospital evaluation of non-transported pediatric patients by a large emergency medical services system. Pediatr Emerg Care. 2001;17(6):421–4. 11753185 10.1097/00006565-200112000-00005

[7] Cone DC Kim DT Davidson SJ . Patient-initiated refusals of prehospital care: ambulance call report documentation, patient outcome, and on-line medical command. Prehosp Disaster Med. 1995;10(1):3–9. 10155403 10.1017/s1049023x0004156x

[8] Zachariah B Bryan D Pepe P et al . Follow‐up and outcome of patients who decline or are denied transport by EMS. Prehosp Disaster Med. 1992;7(4):359–64.

[9] Knight S Olson LM Cook LJ et al . Against all advice: an analysis of out-of-hospital refusals of care. Ann Emerg Med. 2003;42(5):689–96. 14581923 10.1016/S0196064403005249

[10] Seltzer AG Vilke GM Chan TC et al . Outcome study of minors after parental refusal of paramedic transport. Prehosp Emerg Care. 2001;5(3):278–83. 11446543 10.1080/10903120190939797

[11] Satty T Ramgopal S Elmer J et al . EMS responses and non-transports during the COVID-19 pandemic. Am J Emerg Med. 2021;42:1–8. 33429185 10.1016/j.ajem.2020.12.078 PMC7836527

[12] Joy T Bosson N Chang A et al . 86 short-term outcomes and patient perceptions after EMS non-transport during the COVID-19 pandemic. Ann Emerg Med. 2022;80(4):S43–4.

[13] Glober N Hamilton J Montelauro N et al . Safety of an alternative care protocol for EMS non-transport in the COVID-19 pandemic. Prehosp Emerg Care. 2023;27(3):315–20. 35666266 10.1080/10903127.2022.2086652 PMC12203385

[14] Haines CJ Lutes RE Blaser M et al . Paramedic initiated non-transport of pediatric patients. Prehosp Emerg Care. 2006;10(2):213–9. 16531379 10.1080/10903120500541308

[15] Sinclair JE Austin M Froats M et al . Characteristics, prehospital management, and outcomes in patients assessed for hypoglycemia: repeat access to prehospital or emergency care. Prehosp Emerg Care. 2019;23(3):364–76. 30111210 10.1080/10903127.2018.1504150

[16] Coster J O’Cathain A Jacques R et al . Outcomes for patients who contact the emergency ambulance service and are not transported to the emergency department: a data linkage study. Prehosp Emerg Care. 2019;23(4):566–77. 30582719 10.1080/10903127.2018.1549628

[17] Lerner EB Dayan PS Brown K et al . Characteristics of the pediatric patients treated by the Pediatric Emergency Care Applied Research Network’s affiliated EMS agencies. Prehosp Emerg Care. 2014;18(1):52–9. 24134593 10.3109/10903127.2013.836262

[18] Hewes HA Dai M Mann NC et al . Prehospital pain management: disparity by age and race. Prehosp Emerg Care. 2018;22(2):189–97. 28956669 10.1080/10903127.2017.1367444

[19] Topjian AA Raymond TT Atkins D et al . Part 4: Pediatric Basic and Advanced Life Support: 2020 American Heart Association Guidelines for Cardiopulmonary Resuscitation and Emergency Cardiovascular Care. Circulation. 2020;142(16_suppl_2):S469–523. 33081526 10.1161/CIR.0000000000000901

[20] USA Facts Organization . Our Changing Population: Dallas, County, Texas. Available at: https://usafacts.org/data/topics/people-society/population-and-demographics/our-changing-population/state/texas/county/dallas-county?endDate=2020-01-01&startDate=2019-01-01. Accessed January 12, 2023.

[21] Kahalé J Osmond MH Nesbitt L et al . What are the characteristics and outcomes of nontransported pediatric patients? Prehosp Emerg Care. 2006;10(1):28–34. 16418088 10.1080/10903120500373322

[22] Oulasvirta J Salmi H Kuisma M et al . Outcomes in children evaluated but not transported by ambulance personnel: retrospective cohort study. BMJ Paediatr Open. 2019;3(1):e000523. 10.1136/bmjpo-2019-000523 PMC6830473 31750406

[23] Hartka T Vaca FE . Factors associated with EMS transport decisions for pediatric patients after motor vehicle collisions. Traffic Inj Prev. 2020;21(sup1):S60–5. 33119415 10.1080/15389588.2020.1830382 PMC8081732

